# The Objects and Process of Its Manufacture

**Published:** 1921-03-12

**Authors:** 


### DRIED MILK.

### I. The Objects and Process of its Manufacture.

By A MEDICAL OFFICER OF HEALTH.

It is one of the commonplaces of food organisation that the general population must have a plentiful and good supply of milk. The use of milk is recorded in our oldest historical documents, and its distribution is world-wide both in civilised and uncivilised communities. As an article of diet it is especially important for children and invalids, and it has also its use for adults, for though there are medical men who question its utility for these, they are in a very small minority. The writer can mention districts especially in certain parts of Ireland where creameries have had the effect of limiting the use of milk for ordinary farm labourers to the detriment of their health.

The first thought which presents itself is that milk contains eighty-seven per cent. of water. The presence of so large an amount adds very seriously to the difficulties of transport both from a health and economic point of view. On the other hand, the constitution of milk is complicated, and though we can make a rough chemical analysis of its constituents, we are completely in the dark as to how they are combined, as to how the minerals are incorporated with the protein, and what relations both have with the enzymes which are known to exist in raw milk. Early preservatives were used with the idea of preventing bacterial growth and at the same time not interfering with its enzymes, or so-called "vital" qualities; these, however, were soon abandoned, because it was found that the remedy was worse than the disease, and that more harm might be expected to accrue from the preservatives than from the bacteria, especially as it was found that those employed had only a slight retarding influence on the onset of decomposition.

With the abandonment of such preservatives the question of heating naturally came to be investigated. On the first discovery of the enzymes in milk it was found that heating to boiling-point destroyed the enzymes and in some mysterious way altered the proteins. Later, on the discovery of vitamines, it was assumed that these were destroyed by heat, but further investigation by Dr. Lane-Claypon and others showed that it very largely depends on the temperature and length of exposure whether any harm is done to these substances. One advantage, however, in cooking milk is that practically all bacterial life is destroyed, and this may well be set off against the disadvantages, many of them imaginary or theoretical, which accrue to the process of cooking.

That there are advantages from heating milk through the destruction of bacterial life is unquestioned, if one considers for a moment the many diseases which may be conveyed by milk, either through actual disease of the cow—such as tuberculosis—or through accidental contamination of the milk by pathogenic bacilli after the milk has left the cow. Here we are on positive ground, whereas the harm said to result from heating rests on much more slender evidence, and is rather based on theoretical conceptions.

Assuming, then, for the moment that judicious heating of milk—that is, not too high, and not too prolonged—does little harm to its nutritive qualities, the next question is, Can heat be utilised to get rid of the water, and how much water can with safety be got rid of?

According to a Local Government Board report on dried milk published in 1918 dessicated milk seems to have been known of in 1868, and various attempts were made to produce it between this date and 1900, when Robert Stauff, of Posen, produced a pure milk powder; but all these milk powders were mixed with cane sugar or some other substance so as to render them easily powdered. During the early years of the present century many processes were patented which differ in details and efficiency, but the methods practically resolve themselves into two—viz., one by which the milk comes in contact with steam-heated rollers for a very short period numbered by seconds, and another in which it is sprayed into hot air and rapidly dried by it.

In the first process there are two heated steel rollers running in opposite directions and almost touching, and as they revolve inwards towards one another the wedge-shaped space, with its apex formed by the approximation of the rollers and its base pointing upwards, is filled with milk from a receptacle suspended above, where it soon boils, leaving a thin white film of dried milk on the roller surface, which is rapidly carried underneath and round to be automatically scraped off by two sharp knives. In another form of machine the milk is first condensed by a small roller, and then passed on to a single large roller, where the drying of the film is facilitated by a blast of hot air sent over its surface.

In the second process mentioned above the milk is first separated; then pasteurised, remixed, and condensed *in vacuo* to a syrupy consistence at a temperature below boiling-point. Finally, it is forced through a fine nozzle in the form of spray along with hot air into a chamber where the droplets of milk quickly become dried and fall to the bottom as a fine white powder. The milk powder dried by this process is very fine, and when reconstituted by mixing with water gives a homogeneous fluid with little separation of the fat. In the roller process the milk when reconstituted often shows droplets of butter fat floating on the surface. The powder, however, made by the spraying process does not seem to keep so well as that made by the hot rollers.

(*To be continued.*)

